# Cerebral Venous Sinus Thrombosis Revealing ALK-Positive Anaplastic Large-Cell Lymphoma in a Patient with Inherited Thrombophilia and Concomitant Infection: Case Report and Narrative Review

**DOI:** 10.3390/life16081329

**Published:** 2026-08-13

**Authors:** Traian Flavius Dan, Alexandra Timeea Pis, Ana-Maria-Smaranda Ulucean, Alexandra Copil, Razvan Bertici, Adelina Miron, Andreea Mihaela Borz, Georgiana Munteanu, Nicoleta Iacob, Ioana Ionita, Silviana Nina Jianu, Dragos Catalin Jianu

**Affiliations:** 1 First Division of Neurology, Department of Neurosciences, Victor Babes University of Medicine and Pharmacy, E. Murgu Sq., no.2, 300041 Timisoara, Romania; traian.dan@umft.ro (T.F.D.); razvan.bertici@umft.ro (R.B.); munteanu.georgiana@umft.ro (G.M.); jianu.dragos@umft.ro (D.C.J.); 2 Centre for Cognitive Research in Neuropsychiatric Pathology (NeuroPsy-Cog), Department of Neurosciences, Victor Babes University of Medicine and Pharmacy, 156 L. Rebreanu Ave., 300736 Timisoara, Romania; 3 First Department of Neurology, Pius Brînzeu Clinical Emergency County Hospital, 156 L. Rebreanu Ave., 300736 Timisoara, Romania; timeea.pis@rezident.umft.ro (A.T.P.); ana-maria.ulucean@umft.ro (A.-M.-S.U.); adelina.miron@rezident.umft.ro (A.M.); 4 Gemeinnützige Salzburger Landeskliniken Betriebsgesellschaft mbH, Nuklearmedizin, Müllner Hauptstraße 48, 5020 Salzburg, Austria; andreea_tanase1005@yahoo.com; 5 Department of Multidetector Computed Tomography and Magnetic Resonance Imaging, ScanExpert Diagnostic Imaging Centre, 300218 Timisoara, Romania; nicoiacob@yahoo.co.uk; 6 Division of Hematology, Department V, “Victor Babes” University of Medicine and Pharmacy, E. Murgu Sq., no.2, 300041 Timisoara, Romania; ionita.ioana@umft.ro; 7 Department of Ophthalmology, “Dr. Victor Popescu” Military Emergency Hospital, 7 G. Lazar Ave., 300080 Timisoara, Romania; silvianajianu@yahoo.com

**Keywords:** cerebral venous sinus thrombosis (CVST), ALK-positive anaplastic large cell lymphoma (ALCL), hereditary thrombophilia, diagnostic delay, multidisciplinary management, cancer-associated thrombosis

## Abstract

Cerebral venous sinus thrombosis (CVST) is an uncommon cerebrovascular disorder with heterogeneous manifestations and may occasionally precede the diagnosis of an underlying malignancy. We report the case of a 24-year-old man who presented with recurrent fever, headache, pharyngodynia, and systemic inflammation initially attributed to a sinonasal or odontogenic infectious process. He subsequently developed binocular horizontal diplopia, left abducens nerve palsy, papilledema, severe headache, and nausea. Neuroimaging demonstrated extensive CVST involving the left internal jugular vein, bilateral transverse sinuses, and superior sagittal sinus, without ischemic, hemorrhagic, or tumoral brain parenchymal lesions. Thrombophilia testing identified heterozygous prothrombin G20210A as the only established inherited thrombophilic factor. Despite initial neurological stabilization, the patient developed a rapidly recurrent frontal calvarial, epicranial, and cranio-dural lesion extending toward the superior sagittal sinus, without brain parenchymal involvement or imaging evidence of leptomeningeal disease. Initial morphological assessment suggested Langerhans cell histiocytosis. However, comprehensive histopathological and immunohistochemical reassessment demonstrated diffuse strong CD30 expression, nuclear and cytoplasmic ALK positivity, CD43 expression, and focal epithelial membrane antigen and granzyme B positivity, while CD1a and S100 were negative. These findings established the diagnosis of systemic ALK-positive anaplastic large cell lymphoma with secondary extra-axial cranio-dural involvement. Systemic staging demonstrated disseminated nodal disease and a noncontiguous cranio-dural extranodal lesion, consistent with stage IV disease. Treatment with anticoagulation and six cycles of brentuximab vedotin combined with cyclophosphamide, doxorubicin, and prednisone resulted in a favorable neurological and oncological outcome, with no metabolically active or residual enhancing disease on follow-up imaging. This case emphasizes the importance of continued etiological investigation in young patients with extensive CVST and an atypical clinical course, even when plausible infectious and inherited thrombotic risk factors coexist.

## 1. Introduction

Cerebral venous sinus thrombosis (CVST) is an uncommon, potentially life-threatening cerebrovascular disorder [1]. While its estimated annual incidence is low (3 to 4 cases per million), CVST disproportionately affects younger individuals, particularly those with underlying prothrombotic states, hereditary thrombophilia, or individuals exposed to hormone-related prothrombotic factors, including oral contraceptive use, pregnancy, and the puerperium [2,3].

Due to the marked anatomical variability of the cerebral venous system, comprising the superficial and deep venous compartments as well as the dural sinuses, the clinical presentation of CVST is highly heterogeneous. Given its frequently nonspecific neurological manifestations, CVST requires a high level of clinical vigilance among physicians from different specialties, including neurologists, infectious disease experts, and oncologists. Additionally, prompt use of advanced neuroimaging modalities, such as computed tomography venography (CTV), contrast-enhanced magnetic resonance imaging (MRI), and magnetic resonance venography (MRV), is essential for timely and accurate diagnosis [3,4,5,6].

The etiology of CVST is multifactorial and often reflects an interplay between systemic prothrombotic conditions, including hereditary or acquired thrombophilia, malignancy, systemic infection, and chronic inflammatory or autoimmune disease, and local precipitating factors, such as head and neck infections, cranial trauma, neurosurgical procedures, or other regional inflammatory processes [7,8,9,10,11]. The International Study on Cerebral Vein and Dural Sinus Thrombosis (ISCVT) showed that more than 85% of patients had at least one identifiable risk factor and that inherited thrombophilias were identified in a notable proportion of patients [12]. Variations in venous anatomy and drainage patterns may complicate neuroimaging interpretation and contribute to diagnostic uncertainty [4,11,13,14]. In the presence of coexisting conditions, such as sources of infection or inflammation, clinical manifestations may be difficult to interpret, making the distinction between an infectious precipitant and an underlying hematologic malignancy a significant diagnostic challenge [15,16,17,18,19].

This case report aims to highlight the diagnostic complexity of CVST in young patients with multiple potential prothrombotic and inflammatory factors. In particular, it emphasizes the challenges of distinguishing between local infectious precipitants, hereditary thrombophilia, and occult malignancy as contributing causes of CVST. The report also underscores the importance of timely neuroimaging, comprehensive etiological assessment, and a multidisciplinary diagnostic approach in patients presenting with persistent systemic symptoms and atypical neurological manifestations.

## 2. Case Presentation

We present the case of a 24-year-old man with no relevant personal or family medical history, no known predisposing risk factors, no history of tobacco or alcohol use, and no long-term medication use. In May 2023, he presented to his general practitioner with recurrent headaches, pharyngodynia, and persistent fever that had begun several days earlier. His symptoms were initially attributed to an acute upper respiratory tract infection.

### 2.1. Initial Presentation

He was referred to the pulmonology department for further evaluation and was subsequently admitted. Throughout his hospital stay, his vital signs remained within normal limits, except during recurrent febrile episodes, when his temperature reached approximately 38 °C every 6–8 h and decreased only transiently following antipyretic administration. The relapsing fever and headaches, together with increasing fatigability and generalized arthralgia, were accompanied by a gradual deterioration in his overall clinical condition.

Further evaluation of the treatment-resistant relapsing fever and headaches raised the possibility of a sinonasal or odontogenic source of infection. Otorhinolaryngological examination revealed left maxillary sinusitis, while dental assessment identified multiple carious lesions as potential odontogenic foci. Consequently, dental extractions and professional oral hygiene procedures were performed. Following these interventions, the patient’s general condition improved and his symptoms subsided, allowing him to be discharged.

**Figure 1:**
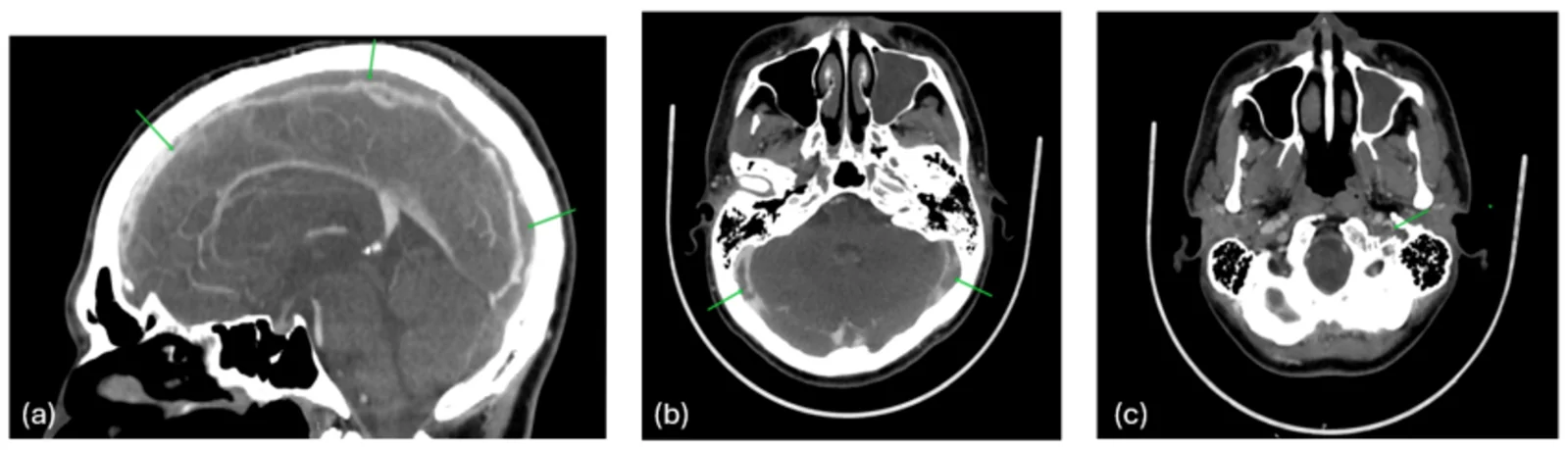
Cerebral Angio-CT revealed thrombosis (arrows) in the superior sagittal sinus (**a**), in the transverse sinuses (**b**) and in the left internal jugular vein (**c**).

However, the febrile syndrome recurred shortly after discharge (by August 2023), followed by the acute onset of binocular horizontal diplopia, left convergent strabismus, severe headache, and nausea. He was urgently referred to the emergency department for neurological assessment. On examination, he was conscious, cooperative, and oriented to time and place, with no signs of meningeal irritation. Neurological examination revealed an isolated left abducens nerve palsy, without other significant focal deficits. Given the suspicion of an acute cerebrovascular event, emergency non-contrast computed tomography (CT) and CT angiography (CTA) were performed, as magnetic resonance imaging was not immediately available in the emergency setting. CTA demonstrated multifocal thrombotic filling defects involving the left internal jugular vein, transverse sinuses, and superior sagittal sinus. No arterial abnormalities, aneurysmal dilatations, arteriovenous malformations, or other pathological cerebral parenchymal changes were identified (Figure 1).

Following admission an ophthalmological examination (fundoscopy) revealed bilateral optic disc swelling, with elevated hyperemic discs, blurred margins, loss of physiological cupping, venous engorgement, and partial obscuration of the peripapillary vessels, consistent with papilledema.

**Figure 2:**
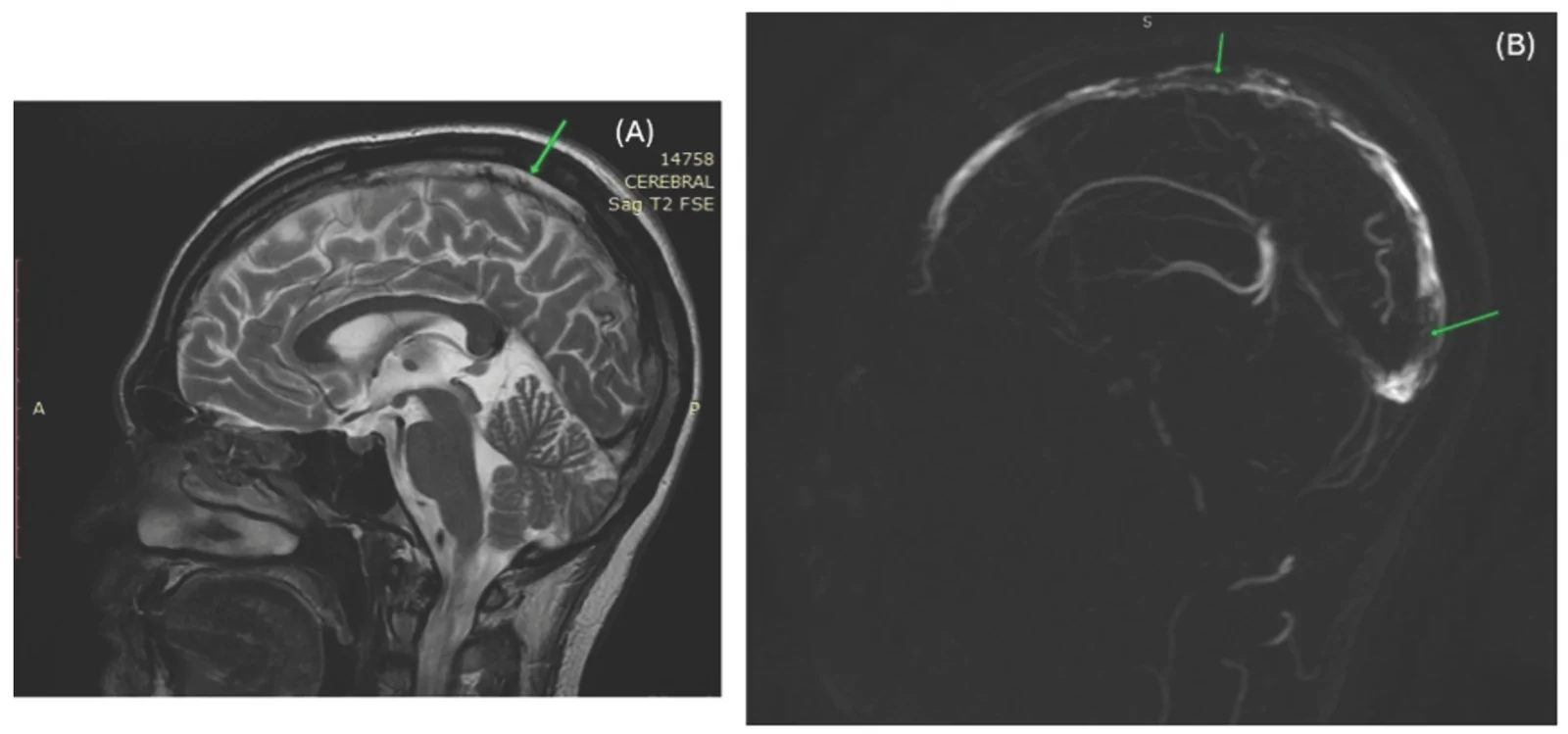
Cranial magnetic resonance imaging. Sagittal T2-weighted imaging showed increased signal intensity within the superior sagittal sinus, suggestive of venous thrombosis (arrow) (**A**), while 2D TOF MRV demonstrated areas of absent flow corresponding to superior sagittal sinus thrombosis (**B**). The letters A and P indicate anterior and posterior orientation.

Contrast-enhanced brain MRI and two-dimensional time-of-flight magnetic resonance venography (2D TOF MRV) further confirmed extensive CVST, showing near-complete occlusion of both transverse sinuses. At the level of the superior sagittal sinus, alternating areas of residual partial flow and complete flow void were observed, with additional low-intensity residual flow in cortical venous pathways converging toward the superior sagittal sinus (Figure 2).

### 2.2. Unexpected Complication

Following partial neurological stabilization, the patient’s clinical course remained atypical and progressively complex. Within two weeks, he developed a fluctuating pseudotumoral epicranial lesion in the frontal region, associated with discontinuous erosion of both the inner and outer cranial tables, requiring neurosurgical biopsy and lesion excision. The lesion showed a rapidly progressive behavior, with local recurrence, after less than 2 months, requiring further neurosurgical management. Subsequent intervention consisted of ablation of the recurrent cranio-dural tumor, which extended toward the superior sagittal sinus, followed by reconstruction of the parietal defect using a scalp graft.

**Figure 3:**
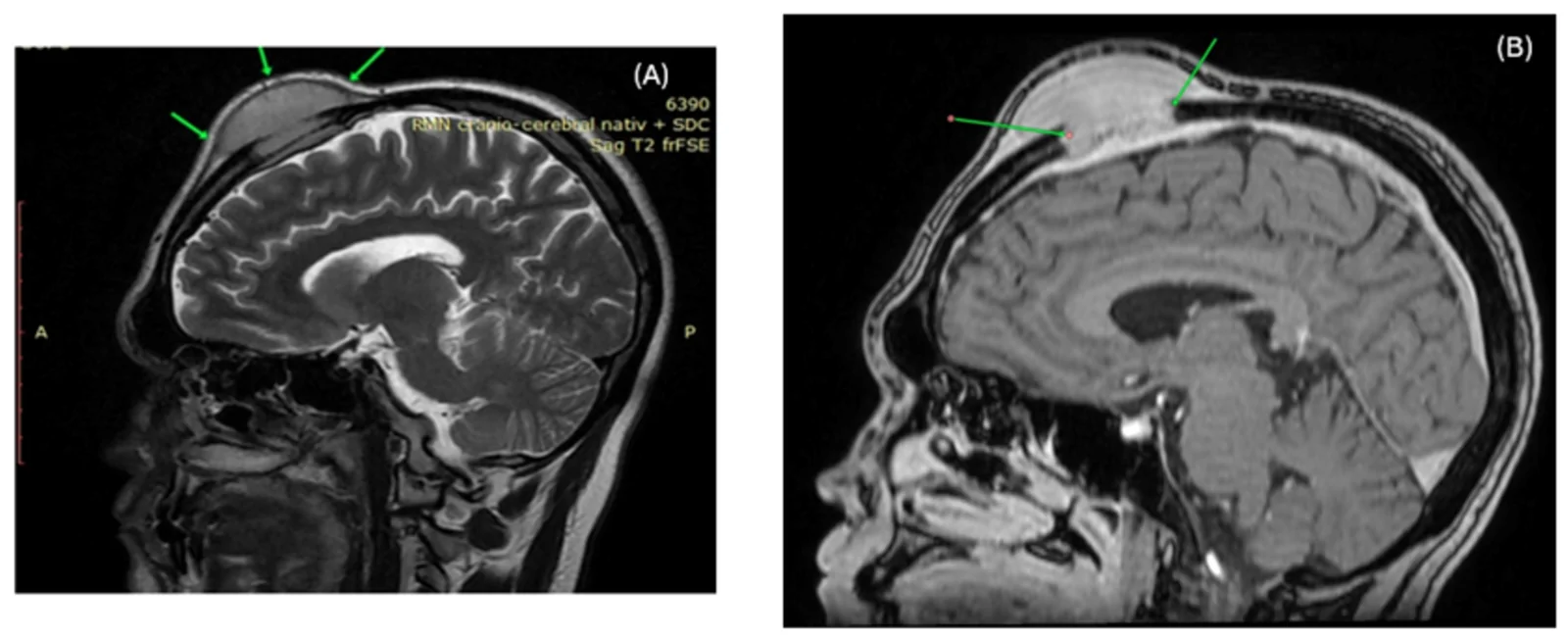
Brain MRI showed a frontal osteolytic bone tumor extended epicranially and subdurally (**A**) with significant contrast uptake, infiltrating the superior sagittal sinus (**B**) but without brain parenchymal involvement. The letters A and P indicate anterior and posterior orientation.

Head MRI revealed a significant increase in the volume of the frontal osteolytic bone lesion, with epicranial and subdural extension and infiltration of the superior sagittal sinus but without evidence of brain parenchymal involvement (Figure 3).

**Figure 4:**
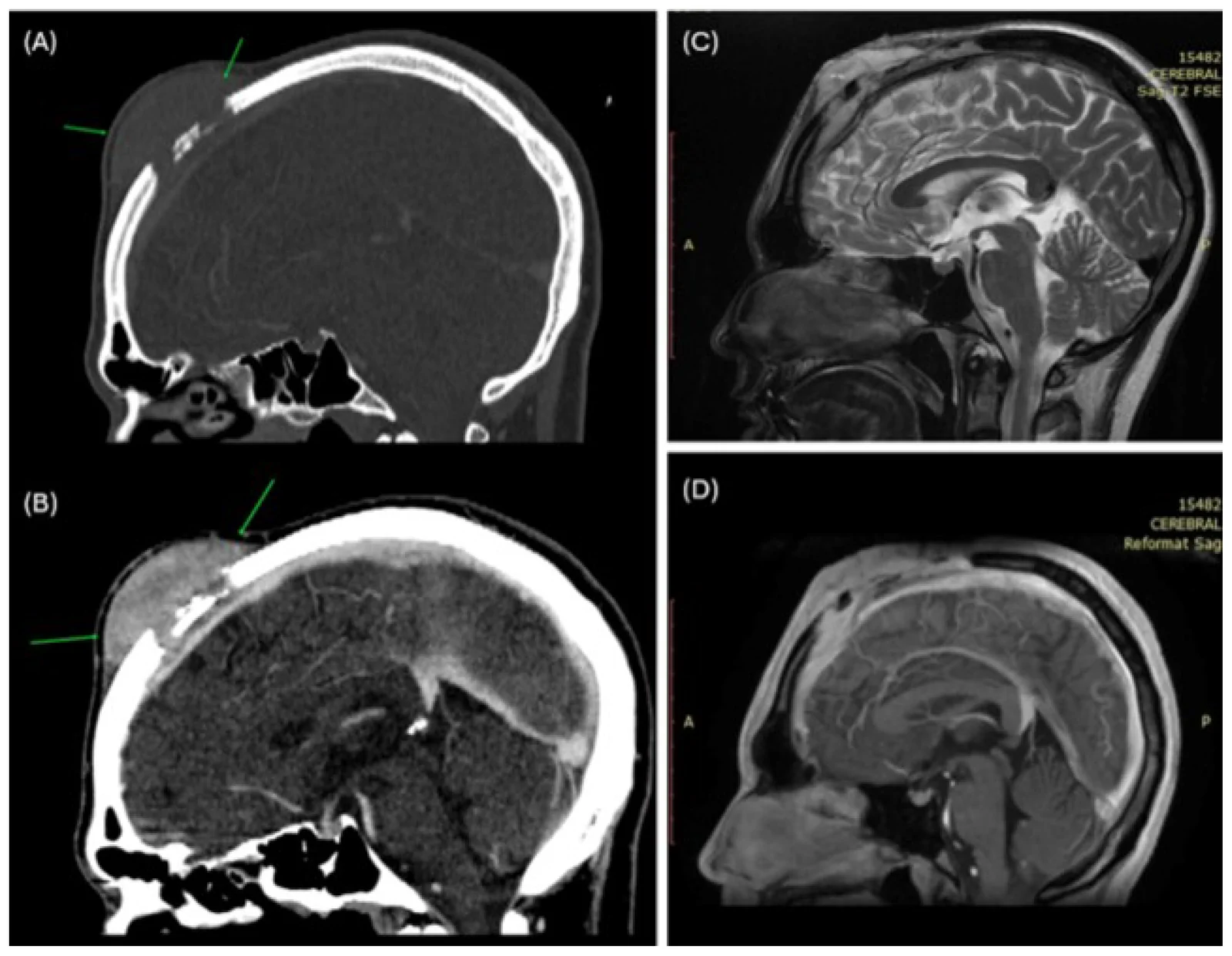
Angio-CT with venous time highlighted the frontal osteolytic tumor extended epicranially and invasive in the superior sagittal dural sinus (**A**,**B**). Postoperative brain MRI showed the dimensional increase in the frontal bone tumor (**C**,**D**). The letters A and P indicate anterior and posterior orientation.

Venous-phase CT angiography performed at admission to the Neurosurgery Department demonstrated a frontal osteolytic lesion with epicranial extension and invasion of the superior sagittal sinus. Several weeks after the surgical procedure, brain MRI was performed showing an increase in the size of the frontal bone lesion (Figure 4).

**Figure 5:**
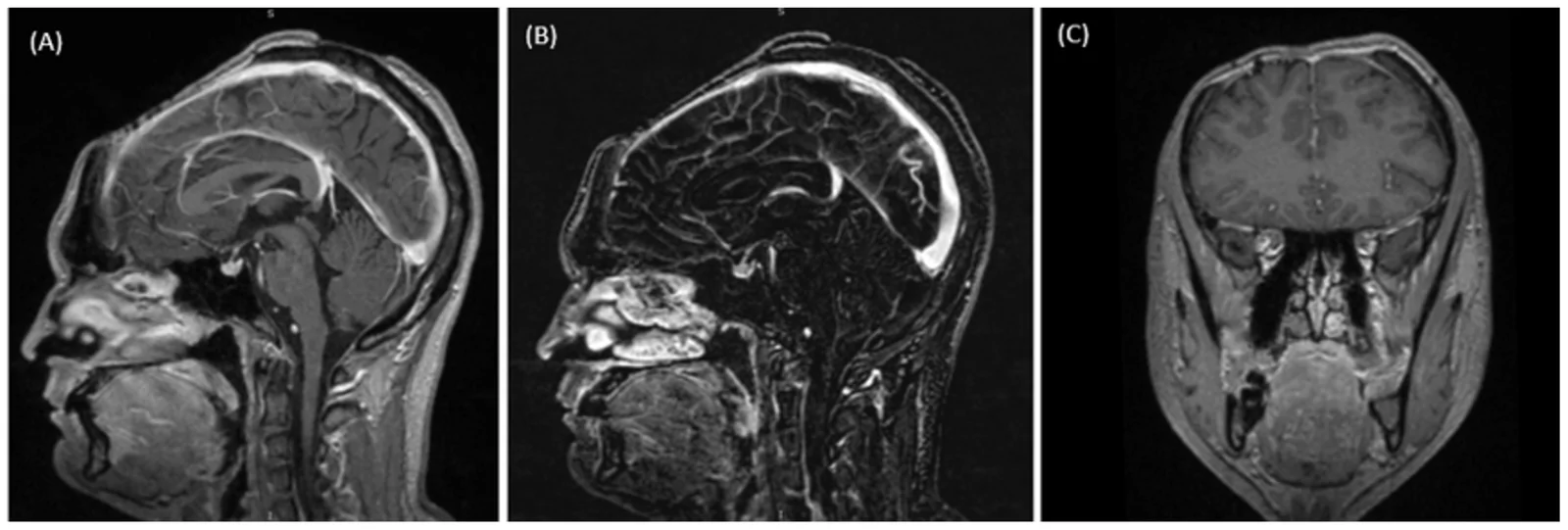
Follow-up contrast-enhanced brain MRI. Sagittal postcontrast 3D T1-weighted image (**A**), sagittal subtraction image (**B**), and coronal reformatted postcontrast image (**C**) demonstrate complete radiological resolution of the previously identified frontal lesion, with no residual or recurrent contrast-enhancing tumoral tissue.

Figure 5 shows follow-up brain MRI demonstrating complete radiological resolution of the previously identified tumoral lesion, with no residual enhancing tissue at the affected site, findings consistent with a favorable therapeutic response.

### 2.3. Laboratory Data

In the context of persistent fever, a marked inflammatory syndrome, and the subsequent development of CVST, an extensive laboratory and genetic evaluation was performed. C-reactive protein levels remained elevated, increasing from 51 mg/L to 85 mg/L, while the erythrocyte sedimentation rate was 80 mm/h. The leukocyte count remained within the normal range at 8.63 × 10^3^/µL. Repeated microbiological investigations, including nasopharyngeal testing, sputum examination for Mycobacterium tuberculosis, viral serologies, urine cultures, and serial blood cultures, yielded negative results. Consequently, the diagnosis of an otorhinolaryngological infectious process was based primarily on the clinical presentation, imaging findings, and associated inflammatory response rather than on microbiological confirmation.

After the onset of neurological symptoms, lumbar puncture was performed to exclude a central nervous system infection, given the concern for possible contiguous spread from the sinonasal or odontogenic infectious focus. The cerebrospinal fluid was clear and showed a white blood cell count of 33 cells/µL, a red blood cell count of 19 cells/µL, an elevated protein concentration of 0.70 g/L, normal glucose concentration, and an opening pressure of 24 cm H_2_O. Bacterial and fungal cultures were negative, providing no microbiological evidence of central nervous system infection. CSF cytology and flow cytometry were not performed because the lumbar puncture preceded any clinical suspicion of hematological malignancy and was not repeated after the diagnosis of ALK-positive ALCL.

In the context of extensive CVST, thrombophilia testing was performed to investigate a possible inherited prothrombotic predisposition. The panel identified heterozygosity for the prothrombin G20210A variant, which was considered the only established inherited thrombophilic factor among the reported findings. Additional findings included the MTHFR C677T and Factor XIII V34L polymorphisms, the homozygous PAI-1 5G/5G genotype, and EPCR gene variants. These findings were reported for completeness but were not interpreted as established independent prothrombotic factors.

### 2.4. Evolving Diagnosis

The patient’s initial presentation was highly nonspecific, consisting mainly of recurrent fever and headache, with few systemic features suggesting an underlying malignancy. His young age, unremarkable medical history, and absence of established major risk factors made a possible locoregional infection a reasonable initial explanation for the clinical episodes. However, the subsequent development of acute neurological manifestations substantially increased the diagnostic complexity.

At the onset of the neurological symptoms, neuroimaging showed extensive CVST but no ischemic or hemorrhagic parenchymal lesion and no detectable intracranial tumoral involvement. In the context of the preceding sinonasal or odontogenic inflammatory process, the thrombosis was initially considered potentially related to the local infection. Nevertheless, because of its unusual extent and distribution in a young patient without conventional thrombotic risk factors, thrombophilia screening was performed. The identification of heterozygous prothrombin G20210A, a recognized but modest inherited thrombophilic factor, was initially considered, together with the locoregional inflammatory process, a plausible explanation for the CVST. CSF findings did not provide significant clarification at this point. The subsequent atypical clinical evolution, however, indicated that these findings did not fully account for the patient’s presentation.

With the emergence of the frontal epicranial cranio-dural lesion and its rapid recurrence after surgical excision, diagnostic attention shifted toward a possible underlying neoplastic process.

The initial biopsy specimen was evaluated at a local pathology laboratory. Morphological examination revealed large atypical cells arranged in a non-cohesive pattern, with abundant pale cytoplasm and irregular, grooved, reniform, or occasionally multinucleated nuclei. In conjunction with the eosinophil-rich inflammatory background and the anatomical location of the lesion, these findings raised suspicion of Langerhans cell histiocytosis. However, definitive immunophenotypic confirmation was not possible because staining for CD1a, langerin, and S100 was unavailable at that center.

Following the rapid recurrence of the lesion and a second surgical excision, the initial diagnosis was reconsidered, and the new specimen was submitted to a reference pathology center for comprehensive histopathological and immunohistochemical assessment. Examination revealed a diffuse proliferation of large pleomorphic lymphoid cells with abundant cytoplasm and irregular, occasionally horseshoe-shaped or reniform nuclei, dispersed among histiocytes and polymorphonuclear inflammatory cells. The neoplastic cells showed diffuse and strong CD30 expression, together with CD43 positivity and focal expression of granzyme B and epithelial membrane antigen. The absence of CD3 and CD20 supported a T-cell/null immunophenotype.

In addition, nuclear and cytoplasmic ALK expression was highly suggestive of an NPM–ALK fusion, while negative CD1a, langerin/CD207, and S100 staining argued against Langerhans cell histiocytosis. Collectively, these findings established the diagnosis of ALK-positive anaplastic large cell lymphoma (ALCL).

Following histopathological confirmation of ALK-positive ALCL, systemic staging was completed. Bone marrow examination showed preserved cellularity with dysmegakaryopoiesis but no evidence of lymphoma involvement. Baseline fluorodeoxyglucose positron emission tomography–computed tomography (FDG PET/CT) demonstrated disseminated metabolically active nodal lesions together with the aggressive frontal calvarial, epicranial, and cranio-dural lesion, which represented the only documented extranodal site. Based on the disseminated nodal involvement and the noncontiguous extranodal cranio-dural lesion, the disease was classified as stage IV ALK-positive ALCL according to the Lugano-modified Ann Arbor staging system.

The acute neurological manifestations, including severe headache, nausea, binocular horizontal diplopia, left convergent strabismus due to abducens nerve palsy, and papilledema were primarily attributed to CVST-related intracranial hypertension. In contrast, the recurrent frontal epicranial mass, calvarial destruction, cranio-dural extension, disseminated nodal lesions, and persistent constitutional inflammatory syndrome were considered manifestations of ALK-positive ALCL. Nevertheless, the recurrent fever and systemic inflammation could not initially be attributed unequivocally to either infection or lymphoma because of the substantial clinical overlap. The case specific differential diagnostic features between Langerhans cell histiocytosis and ALK-positive ALCL are summarized in Table 1.

**Table 1:** Case-specific differential diagnosis between Langerhans cell histiocytosis and ALK-positive ALCL.

Diagnostic Feature	Findings Initially Suggesting Langerhans Cell Histiocytosis ^1^	Findings Supporting ALK-Positive ALCL
Morphology	Non-cohesive large atypical cells with abundant pale cytoplasm and grooved or reniform nuclei	Diffuse proliferation of large pleomorphic lymphoid cells, including cells with horseshoe-shaped or reniform nuclei
Histological context	Eosinophil-rich inflammatory background and calvarial lesion location	Histiocyte- and polymorphonuclear cell–rich background associated with an aggressive, rapidly recurrent lesion
Key immunohistochemistry	CD1a, langerin/CD207, and S100 were unavailable during the initial evaluation; subsequent staining for all three markers was negative	Diffuse strong CD30 and nuclear–cytoplasmic ALK expression, with CD43 positivity and focal EMA and granzyme B expression
Lineage and final interpretation	LCH could not be immunophenotypically confirmed	CD3 and CD20 negativity, interpreted with the overall immunophenotypic profile, supported a T-cell/null phenotype and established ALK-positive ALCL

^1^ The findings presented in the left column reflect the initial provisional morphological interpretation before comprehensive immunohistochemical reassessment and should not be regarded as established or specific features of Langerhans cell histiocytosis.

### 2.5. Management, Evolution and Prognosis

In the acute neurological phase, the patient was promptly managed with therapeutic anticoagulation using low-molecular-weight heparin, initiated after the diagnosis of extensive CVST. Given the clinical evidence of intracranial hypertension, osmotic therapy with intravenous mannitol was initiated promptly. In view of the concomitant suspected infectious and inflammatory process, broad-spectrum antimicrobial treatment was administered concurrently. The immediate therapeutic objective was to prevent thrombus extension, reduce the risk of secondary venous infarction or hemorrhagic complications, and achieve neurological stabilization. Under this treatment, the patient showed partial clinical improvement, with stabilization of the symptoms.

After neurological stabilization, the patient underwent surgical treatment of the left maxillary sinus lesion, consisting of left maxillary antrostomy and endoscopic resection of a cystic lesion occupying the sinus cavity.

Following the oncological diagnosis, the patient was referred to the Hematology Department and received systemic BV-CHP therapy. CNS-directed prophylaxis or intrathecal treatment was not administered because the lymphoma involvement was confined to the calvarium, epicranial soft tissues, and dura, without brain parenchymal involvement or imaging evidence of leptomeningeal disease. The patient received six cycles of BV–CHP at 21-day intervals, consisting of intravenous brentuximab vedotin (126 mg), cyclophosphamide (1388 mg), and doxorubicin (92.5 mg) on day 1, together with oral prednisone (100 mg/day) on days 1–5 of each cycle.

The patient’s subsequent clinical course was favorable with only mild chemotherapy treatment-related neutropenia observed. At the time of the sixth chemotherapy cycle, follow-up PET-CT and brain MRI showed no lesions with pathological metabolic uptake and no evidence of active oncological disease (February 2024).

Following initial treatment with low-molecular-weight heparin during hospitalization, anticoagulation was transitioned to oral apixaban at a dose of 5 mg twice daily upon discharge, after neurological stabilization. Apixaban was continued for six months, including during the administration of BV-CHP chemotherapy. Although evidence specifically evaluating apixaban in malignancy-associated CVST remains limited, DOAC use has been reported in cohorts of patients with cancer-related CVST, and observational data support the safety and efficacy of apixaban in CVST more generally. In addition, apixaban is an established oral treatment option for cancer-associated venous thromboembolism. Its selection in the present case was individualized and was based on its convenient oral administration and favorable efficacy–safety profile. Potential complications, including treatment-related thrombocytopenia, increased bleeding risk, changes in renal or hepatic function, and possible drug–drug interactions, were assessed and closely monitored throughout treatment [20,21].

In October 2024, contrast-enhanced brain MRI revealed no pathological meningeal or cerebral enhancement and no imaging signs suggestive of residual or recurrent lesion activity. During follow-up, the patient demonstrated progressive neurological and systemic stabilization, with no reported recurrence of diplopia, ocular motor impairment, or other additional thrombotic events.

The patient remains under close hematological surveillance, with follow-up evaluations every six months, including clinical examination and PET/CT imaging. Anticoagulation was subsequently discontinued after clinical and radiological reassessment indicated that it was no longer required.

## 3. Discussion

To contextualize the present case, a narrative literature search was conducted in PubMed and Clarivate Web of Science for English-language publications issued between 2004 and 2026. Older foundational publications identified through reference-list screening were also considered when directly relevant to the present case. Search terms addressed CVST in association with malignancy, hematological neoplasms, ALK-positive anaplastic large cell lymphoma, inherited thrombophilia, and infectious triggers, including sinusitis and odontogenic infection. Relevant original studies, case reports, case series, reviews, and clinical guidelines were considered, and the reference lists of selected publications were screened for additional sources.

CVST is an uncommon but clinically relevant cerebrovascular disorder, particularly in young adults, and is characterized by a highly heterogeneous and often misleading presentation [22,23]. Patients may present with headache, papilledema, cranial nerve palsies, seizures, focal neurological deficits, or altered consciousness, while venous infarction and intracranial hemorrhage may occur as complications of impaired venous drainage [3,24]. Because these manifestations are frequently nonspecific and may be preceded by systemic inflammatory or infection-like symptoms, diagnosis can be delayed [25,26,27]. Venous outflow obstruction leads to venous hypertension, impaired cerebrospinal fluid absorption, and intracranial hypertension. Headache is the most frequent symptom, occurring in approximately 70–90% of patients, whereas focal neurological deficits are reported in 30–50% [28].

In the present patient, severe headache, nausea, binocular horizontal diplopia, convergent strabismus, and left abducens nerve palsy were consistent with intracranial hypertension secondary to extensive venous congestion [29,30,31,32]. The neurological phenotype of CVST is influenced by the location and extent of venous occlusion [33]. Superior sagittal sinus thrombosis is commonly associated with intracranial hypertension, whereas transverse sinus involvement, particularly when extending toward the torcular Herophili or internal jugular venous outflow, may be accompanied by abducens nerve palsy and an isolated intracranial hypertension syndrome [4,34].

In the context of clinical signs suggestive of intracranial hypertension, including severe headache, nausea, binocular diplopia, convergent strabismus, and abducens nerve palsy, the ophthalmological assessment played an important diagnostic role by documenting optic disc edema consistent with papilledema. This finding supported the presence of raised intracranial pressure secondary to CVST, in which impaired venous drainage and venous congestion may compromise CSF absorption and promote intracranial hypertension. In line with the observations reported by Park et al., papilledema represents a clinically relevant marker in CVST and may reflect the severity of venous stasis, particularly when susceptibility-weighted imaging demonstrates impaired cerebral venous outflow. Therefore, in the present case, the detection of papilledema integrated the ophthalmological findings with the neuroradiological evidence of extensive venous thrombosis, strengthening the diagnosis of CVST-related intracranial hypertension and emphasizing the need for early fundoscopic evaluation in patients with persistent headache, diplopia, or other signs of raised intracranial pressure [35].

This case also illustrates the diagnostic difficulty of CVST when the initial presentation is dominated by persistent fever, sinonasal involvement, and systemic inflammation. An additional diagnostic challenge in this case was represented by the ability of ALK-positive ALCL to generate a systemic inflammatory phenotype that may closely mimic infection. Persistent fever, elevated inflammatory markers, and only transient or absent response to antibacterial therapy may initially support an infectious diagnosis, potentially delaying the identification of an underlying malignancy. In this context, the recurrent febrile syndrome and negative microbiological findings should be interpreted retrospectively as possible manifestations of an underlying lymphoma-related inflammatory response. Therefore, in patients with persistent systemic inflammation and an atypical or refractory clinical course, hematologic malignancy should remain an important differential diagnosis, even when localized sites of infection appear to be present [18]. In patients whose evolution deviates from a typical infectious course, the emergence of diplopia, persistent headache, or papilledema should prompt urgent neuroimaging. Early diagnosis by CTA, MRI, and venous imaging is essential, as extensive CVST requires rapid anticoagulation and coordinated multidisciplinary care [23,36].

The etiology of CVST is frequently multifactorial [37]. Established risk factors include inherited thrombophilia, infections of the head and neck region, systemic inflammation, malignancy, trauma, neurosurgical procedures, dehydration, pregnancy, puerperium, and oral contraceptive use. In this case, inherited thrombophilia, persistent sinonasal/odontogenic inflammation, and an occult hematological malignancy represented potentially concurrent thrombotic risk factors [38,39,40,41,42,43,44,45,46,47,48]. However, their individual and relative contributions to the development of CVST cannot be determined from a single case.

Malignancy is a major acquired cause of venous thromboembolism. Although deep vein thrombosis and pulmonary embolism are the most common cancer-associated thrombotic events, CVST may also occur, particularly in patients with systemic inflammation, hematological disease, intracranial involvement, or treatment-related endothelial injury. Cancer-associated CVST may result from tumor-driven hypercoagulability, cytokine-mediated endothelial activation, platelet activation, impaired fibrinolysis, direct venous invasion or compression, chemotherapy, corticosteroid exposure, and central venous catheterization [49,50,51].

Recent data support the association between cancer and CVST. Cancer has been identified as a significant risk factor for CVST, particularly within the first year after cancer diagnosis, while hematological malignancies remain relevant even after excluding leukemia. Population-based data also indicate an increased incidence of newly diagnosed cancer after CVST, especially during the first year, suggesting that extensive or unexplained CVST may represent an early manifestation of occult malignancy [49,52,53,54].

In patients with cranial, dural, or leptomeningeal involvement, local venous obstruction and regional inflammatory activation may further amplify thrombotic risk. Reports of CVST associated with central nervous system lymphoma support this mechanism, although the present case represents an unusual cranio-dural presentation rather than a typical primary CNS lymphoma [55,56,57,58].

ALK-positive ALCL is a rare T-cell non-Hodgkin lymphoma, usually affecting younger patients and characterized by ALK expression, most often related to the NPM–ALK fusion. Although venous thromboembolism is well recognized in lymphoma, CVST as a manifestation of ALK-positive ALCL is rarely described. Data from ALK-rearranged solid tumors suggest that ALK-driven malignancies may be associated with increased thrombotic risk, possibly through tissue factor expression, inflammatory activation, and tumor–endothelial interaction. While these findings cannot be directly extrapolated, they support a biologically plausible link between ALK-driven oncogenesis and thrombosis [55,59,60].

In the present patient, the oncological diagnosis was established only after the recurrence of a cranio-dural lesion and repeat histopathological and immunohistochemical assessment. This delayed confirmation reflects the diagnostic complexity of rare ALCL presentations, especially when the initial picture mimics infection or inflammatory disease. This case emphasizes that recurrent, aggressive, or atypical cranial lesions should prompt repeated tissue evaluation when the initial diagnosis does not fully explain the clinical evolution.

More broadly, rare tumors of the head and neck region may present with nonspecific clinical and imaging features and substantial morphological overlap, underscoring the importance of comprehensive histopathological and immunohistochemical assessment for definitive diagnosis [61,62].

Among the genetic findings identified in this patient, heterozygous prothrombin G20210A represented the only established inherited thrombophilic factor. This variant is associated with increased prothrombin levels and a modest elevation in the risk of venous thromboembolism, including thrombosis at unusual venous sites. By contrast, MTHFR C677T is not recognized as a clinically relevant inherited thrombophilia, and the homozygous PAI-1 5G/5G genotype is not considered a prothrombotic risk genotype. The other reported polymorphisms were likewise not interpreted as established independent contributors to thrombosis. Therefore, the inherited predisposition in this patient was attributed primarily to heterozygous prothrombin G20210A, which was considered a background risk factor rather than the primary cause of the extensive thrombotic event [63,64,65,66].

The infectious/inflammatory context may also have contributed to the thrombotic cascade. Septic thrombosis of dural venous sinuses has historically been associated with infections of the paranasal sinuses, ear, mastoid, orbit, face, and oral cavity, which may facilitate thrombo-inflammatory extension toward the intracranial venous system through valveless and highly interconnected venous pathways [67,68]. In this regard, a case of septic cavernous sinus thrombosis developing in a patient with inherited thrombophilia and acute mastoid infection was reported, emphasizing the synergistic role of local infection and prothrombotic predisposition in intracranial venous thrombosis [15,16,17,30,69,70].

The possible involvement of emissary venous pathways may further explain the anatomical continuity between extracranial inflammatory or thrombotic processes and intracranial dural sinus thrombosis. Jianu et al. described the petrosquamosal sinus as a residual valveless emissary vein connecting the intracranial dural venous sinuses with the extracranial venous system. In their case, a dilated petrosquamosal sinus was identified in a young woman who developed headache, vomiting, diplopia, abducens nerve palsy, and thrombosis of the superior sagittal and lateral sinuses, with clinical improvement and venous recanalization after anticoagulant therapy. This report supports the concept that emissary venous channels may represent clinically relevant extracranial–intracranial communications. In the present case, such pathways may have facilitated thrombo-inflammatory propagation toward the dural venous sinuses; however, the resulting intracranial hypertension should be attributed primarily to dural sinus thrombosis, impaired cerebral venous outflow, venous hypertension, and reduced cerebrospinal fluid absorption [71].

The overlap between infection, thrombophilia, and malignancy represents the central diagnostic challenge of this case. Persistent fever, elevated inflammatory markers, and partial response to antibiotics initially supported an infectious hypothesis. However, recurrent symptoms, negative microbiological findings, extensive CVST, papilledema and the subsequent development of an aggressive cranio-dural lesion argued for a broader etiological reassessment. Thus, the presence of one plausible risk factor should not exclude the search for additional causes when the clinical course is disproportionate or atypical.

Anticoagulation remains the cornerstone of CVST treatment, because it limits thrombus propagation, facilitates recanalization, and reduces recurrence [72,73,74,75,76]. In malignancy-associated CVST, management is more complex due to the competing risks of thrombosis and bleeding, especially in patients with intracranial lesions, cytopenias, chemotherapy, or neurosurgical interventions. Therefore, anticoagulation must be individualized and integrated with treatment of the underlying cause. In this case, favorable evolution was probably achieved through prompt anticoagulation, surgical management of ENT pathology, neurosurgical excision of the recurrent lesion, and systemic oncological therapy [77,78,79,80].

The peculiarity of this case lies in the convergence of extensive CVST in a young adult, inherited thrombophilia, persistent inflammatory syndrome, sinonasal/odontogenic involvement, and delayed diagnosis of stage IV ALK-positive ALCL. Notably, at the time of the initial thrombotic presentation, dedicated systemic oncological imaging had not yet been performed, while neuroimaging demonstrated extensive CVST without detectable intracranial tumoral involvement. Subsequent staging by FDG PET/CT revealed disseminated metabolically active nodal disease, indicating that the systemic lymphoma may already have been present but clinically occult at the time of CVST. Therefore, the thrombotic event preceded the definitive diagnosis of ALK-positive ALCL, although it cannot be concluded that it preceded the biological onset of the malignancy.

Primary CNS ALK-positive ALCL is exceptionally rare with the largest clinicopathological review included only 34 cases, characterized by a median age of 18.5 years, a marked male predominance, and headache as the most frequent presenting symptom. However, that cohort comprised primary CNS-restricted disease and therefore differs from the present case of systemic ALK-positive ALCL with cranio-dural and calvarial involvement, but without brain parenchymal involvement or imaging evidence of leptomeningeal extension. Ju et al. recently reported the first adult case of synchronous CNS and systemic ALK-positive ALCL, presenting with headache, seizures, and an enhancing frontal convexity lesion with broad dural contact. Although this represents the closest published comparator, that case was characterized as synchronous CNS and systemic disease, whereas the present patient had secondary extra-axial cranio-dural involvement without brain parenchymal involvement or imaging evidence of leptomeningeal disease. CVST was not reported in that case. Taken together, the available literature supports interpreting CVST in the present case as an uncommon and potentially multifactorial complication [60,81,82,83,84].

In the present patient, CVST occurred in the setting of several potentially relevant factors, including systemic ALK-positive ALCL, local dural sinus involvement, a preceding infectious–inflammatory process, and heterozygous prothrombin G20210A. However, their individual and relative contributions to the thrombotic event cannot be determined from a single case. Distinguishing lymphoma-related manifestations from those attributable to CVST was also challenging because of their substantial clinical overlap.

Overall, this case illustrates the coexistence of several potential predisposing factors for CVST rather than establishing a combined causal relationship among them. It reinforces the need for comprehensive etiological evaluation in young patients with extensive or unexplained CVST, particularly when persistent fever, systemic inflammation, recurrent cranial lesions, or atypical neurological manifestations are present.

## 4. Conclusions

This case highlights the importance of considering multiple concurrent predisposing factors in young adults with extensive CVST. In this patient, heterozygous prothrombin G20210A, a local infectious or inflammatory process, and occult systemic ALK-positive ALCL represented potentially relevant thrombotic risk factors; however, their individual and relative contributions remain uncertain. When a patient’s clinical course deviates from that expected for an initially suspected infectious condition, clinicians should maintain a high index of suspicion for underlying malignancy. Continued etiological investigation and multidisciplinary management are essential in patients with unexplained or atypical CVST and may facilitate timely diagnosis and favorable long-term outcomes.

## Data Availability

The datasets are not publicly available. Anonymized data may be provided upon request from Traian Flavius Dan.

## Abbreviations

The following abbreviations are used in this manuscript:

A LCL	Anaplastic large cell lymphoma
ALK	Anaplastic lymphoma kinase
CNS	Central nervous system
CT	Computed tomography
CTA	Computed tomography angiography
CTV	Computed tomography venography
CVST	Cerebral venous sinus thrombosis
ENT	Ear, nose, and throat/Otorhinolaryngology
EPCR	Endothelial protein C receptor
ISCVT	International Study on Cerebral Vein and Dural Sinus Thrombosis
MRV	Magnetic Resonance Venography
MRI	Magnetic resonance imaging
MTHFR	Methylenetetrahydrofolate reductase
NPM-ALK	Nucleophosmin–anaplastic lymphoma kinase fusion
PAI-1	Plasminogen activator inhibitor-1
PET-CT	Positron emission tomography–computed tomography
